# Impact of Acid-Base Status on Mortality in Patients with Acute Pesticide Poisoning

**DOI:** 10.3390/toxics9020022

**Published:** 2021-01-23

**Authors:** Hyo-Wook Gil, Min Hong, HwaMin Lee, Nam-jun Cho, Eun-Young Lee, Samel Park

**Affiliations:** 1 Department of Internal Medicine, Soonchunhyang University Cheonan Hospital, Cheonan 31151, Korea; hwgil@schmc.ac.kr (H.-W.G.); chonj@schmc.ac.kr (N.-j.C.); eylee@schmc.ac.kr (E.-Y.L.); 2 Department of Software Convergence, Soonchunhyang University, Asan 31538, Korea; mhong@sch.ac.kr (M.H.); leehm@sch.ac.kr (H.L.)

**Keywords:** acid-base imbalance, poisoning, pesticides, mortality, principal component analysis

## Abstract

We investigated clinical impacts of various acid-base approaches (physiologic, base excess (BE)-based, and physicochemical) on mortality in patients with acute pesticide intoxication and mutual intercorrelated effects using principal component analysis (PCA). This retrospective study included patients admitted from January 2015 to December 2019 because of pesticide intoxication. We compared parameters assessing the acid-base status between two groups, survivors and non-survivors. Associations between parameters and 30-days mortality were investigated. A total of 797 patients were analyzed. In non-survivors, pH, bicarbonate concentration (HCO_3_^−^), total concentration of carbon dioxide (tCO_2_), BE, and effective strong ion difference (SIDe) were lower and apparent strong ion difference (SIDa), strong ion gap (SIG), total concentration of weak acids, and corrected anion gap (corAG) were higher than in survivors. In the multivariable logistic analysis, BE, corAG, SIDa, and SIDe were associated with mortality. PCA identified four principal components related to mortality. SIDe, HCO_3_^−^, tCO_2_, BE, SIG, and corAG were loaded to principal component 1 (PC1), referred as total buffer bases to receive and handle generated acids. PC1 was an important factor in predicting mortality irrespective of the pesticide category. PC3, loaded mainly with pCO_2_, suggested respiratory components of the acid-base system. PC3 was associated with 30-days mortality, especially in organophosphate or carbamate poisoning. Our study showed that acid-base abnormalities were associated with mortality in patients with acute pesticide poisoning. We reduced these variables into four PCs, resembling the physicochemical approach, revealed that PCs representing total buffer bases and respiratory components played an important role in acute pesticide poisoning.

## 1. Introduction

Pesticides are a large group of heterogeneous chemicals that include a wide range of products, such as insecticides, herbicides, fungicides, and rodenticides [1]. Self-poisoning with pesticides is a global suicide burden and one of the important problems in intensive care unit (ICU) care [2]. Compared to other chemical poisonings, pesticide poisoning is more fatal. Acid-base and electrolyte abnormalities from various causes occasionally develop in patient with acute pesticide poisoning [3,4,5]. Pesticide poisoning can damage organs, including causing cardiovascular collapse, which results in the accumulation of acids. Additives or the chief ingredient could act as anions after pesticides spread throughout the circulation. For example, ethylene glycol and methanol are often used as additives to pesticides, and their metabolites may cause acidosis. Regardless of heterogenous pesticides, electrolyte and acid-base parameters are good predictors reflecting the severity of pesticide poisoning and patient mortality. A few studies have reported the association between anion gap (AG) and mortality in ICU patients and those with pesticide poisoning [3,6,7]. We previously reported that about 33.6% of the patients with acute pesticide poisoning had acid-base disorders [3]. However, paraquat poisoning accounted for the majority (over 85%) of the deaths. The use of paraquat is currently banned in most countries, and this study is of great significance because the pattern of pesticide poisoning has changed [8].

The three approaches are used to assess acid-base disturbances, the physiological [9], base excess (BE) [10], and physicochemical approaches [11]. The physiological approach is assumed to be solely determined by the balance between arterial carbon dioxide tension and the plasma bicarbonate concentration (HCO_3_^−^). BE refers to the dose of base required to adjust the pH back to 7.40 while correcting the arterial carbon dioxide tension to 40 mmHg. Further refinement of these approaches includes AG with and without a correction for hypoalbuminemia (corrected AG, corAG) to define whether excessive anions other than chloride (Cl^−^) and bicarbonate are present [12]. A more contemporary, quantitative approach to acid-base analysis, so called Stewart’s acid-base approach, shows that pH is not simply determined by the [H^+^] and [HCO_3_^−^] (as in the Henderson–Hasselbach approach) but involves the interactions of other variables, three of which are independent variables that control acidity [13]. The Stewart method is based on a physicochemical model, which recognizes that three independent factors affect the acid-base status in mammals, changes in strong ion difference (SID), the partial pressure of CO_2_ (pCO_2_), and the total weak acids and their conjugate bases (total concentration of weak acids, A_Tot_) [14]. Acid-base problems have traditionally been analyzed using a bicarbonate-based approach, which has been criticized for some of its limitations, such as the inability to completely diagnose complex patients [15,16]. However, most studies showed that the physicochemical approach was not superior to other approaches in assessing critically ill patients [17,18].

The parameters assessing acid-base disorders, including AG, SID, strong ion gap (SIG), and BE, are calculated from measured electrolyte concentrations. Although blood gas analyzers measure pH, pCO_2_, and the partial pressure of O_2_ (pO_2_), HCO_3_^−^ and BE are calculated from the blood gas analyzer measurements [10]. SID means the difference between strong cations and anions, in which strong ions are derived from ions fully-dissociated at the pH of body fluids. SIG is calculated by the difference between apparent SID (SIDa) and effective SID (SIDe), representing the presence of an abnormal anion [11,16]. Thus, there are inevitable correlations between electrolytes and the markers representing the status of acid-base disorders. Because of this correlation between independent variables, the collinearity makes traditional regression model inappropriate. Principal component analysis (PCA) is an analytic technique to reduce the dimensionality of big data, thus increasing the interpretability of data by creating new variables with maximized variance, the so-called principal components (PC) [19]. A few variables can be made by mathematical projections from the larger original data set [20]. Therefore, we investigated the changes in acid-base status based on physiologic, BE-based, and physicochemical approaches, according to the pesticide classification. In addition, we investigated the clinical impact of the various acid-base approaches on the mortality of patients with acute pesticide poisoning and the mutual intercorrelated effect using PCA.

## 2. Materials and Methods

### 2.1. Study Population and Design

We conducted a large-scale retrospective cohort study at Soonchunhyang University Cheonan Hospital. Between January 2015 and December 2019, patients admitted to the Institute of Pesticide Poisoning at Soonchunhyang University Cheonan Hospital were enrolled. All the patients were admitted due to intentional poisoning by ingestion with suicidal attempts. The present study was reviewed and approved by Soonchunhyang University Cheonan Hospital’s Investigational Review Board. The requirement for informed consent was waived because of the retrospective design of the study. This study was conducted in accordance with the Declaration of Helsinki.

Demographic variables, such as the age and sex of the patients, were recorded by the physicians on standardized data collection forms. We surveyed the parameters associated with acid-base balance, including pH, pCO_2_, pO_2_, HCO_3_^−^, the total concentration of carbon dioxide (tCO_2_), BE, corAG, and chloride concentration, and other laboratory data. The time difference between the patient’s pesticide exposure and arrival at Soonchunhyang University Cheonan Hospital was recorded by reviewing the patient’s history. The amount of pesticide ingested was estimated from the number of swallows, where one mouthful was considered 20 mL. Paraquat poisoning was excluded because of the high fatality rate. For the laboratory data, we used only data from the patient’s first day of admission for statistical analysis.

The study protocol was reviewed and approved by the Institutional Review Board (IRB) of Soonchunhyang University Cheonan Hospital (Cheonan, Korea) (IRB-No: 2020-02-016). This study was conducted in accordance with principles of the Declaration of Helsinki. The need for informed consent was waived because of its retrospective study design.

### 2.2. Covariates

Initial vital signs upon arrival at the emergency room (ER) were collected. The Glasgow Coma Scale (GCS) score of the worst status within a day of the hospital stay was selected. In intubated patients, an estimated verbal GCS score was calculated based on a previous study as follows: estimated verbal GCS = (2.3976) × (motor GCS × (−0.9253)) + (eye GCS × (−0.9214)) + ((motor GCS)^2^ × (0.2208)) + ((eye GCS)^2^ × (0.2318)) [21]. The patients’ clinical and demographic data, including body mass index (BMI), past medical history of hypertension and diabetes, smoking and alcohol history, the pesticide category, and the estimated ingestion amount were screened by reviewing the electronic medical records (EMRs). Acute physiology and chronic health evaluation (APACHE) II scores were calculated based on the baseline laboratory and clinical data [22].

To calculate the physicochemical acid-base parameters, we used generally accepted formulas and defined the SIDa, SIDe, SIG, and A_Tot_ as follows: (1) SIDa = [Na^+^, mEq/L] + [K^+^, mEq/L] − [Cl^−^, mEq/L]; (2) SIDe = [HCO_3_^−^, mEq/L] + [Alb^−^] + [Pi^−^] where: [Alb^−^] = [Alb, g/L] × [(0.123 × pH) − 0.631] and [Pi^−^] = [Pi, mmol/L] × [(0.309 × pH) − 0.469]; (3) SIG = SIDa − SIDe; and (4) A_Tot_ = 2.7 × [Alb^−^, g/dL] + 0.6 × [Pi, mg/dL] [11,16]. The AG was defined as: AG = [Na^+^, mEq/L] − ([Cl^−^, mEq/L] + [HCO_3_^−^, mEq/L]); then, the corAG was derived by an equation: corAG = AG + 0.25 × (4.0 − measured albumin, g/L).

### 2.3. Statistical Analysis and End Point

All statistical analyses were performed using R version 4.02 (The R Foundation for Statistical Computing, Vienna, Austria). Categorical variables are expressed as counts (percentage). Continuous variables are presented as the mean ± SD or median (interquartile ranges), as appropriate. Student’s *t*-test or Mann–Whitney U test was used for comparison between two groups based on the distribution. Comparisons between multiple groups were performed using one-way ANOVA or the Kruskal-Wallis test, as appropriate. Pearson’s Chi-squared test or Fisher’s exact test was used for categorical variables. The normality of distribution was tested using the Shapiro–Wilk test. A *p*-value of less than 0.05 was considered to have statistical significance. To find associations with mortality within 30 days, the logistic regression model was used. The patients who died within 30 days were classified as non-survivors, and the others were referred to as survivors. Logistic regression models were used to investigate the association of parameters assessing acid-base status and PCs with 30 day mortality. In multivariable analysis, variables with a *p*-value of <0.05 were input into the multivariable model.

Because of the high degree of correlation between parameters assessing acid-base status, PCA was used to combine the parameters to reduce the number of PCs. PCA is a technique to analyze multivariate variables in which observations are represented by several inter-correlated variables. Its goal is to extract a smaller set of variables, which capture most information of the original variables, and to compress the size of data [23]. PCA was done using the R package “psych”. PCs were acquired by shearing of the original variables to the maximizing variance following eigenvectors. Loading, the proportion of original variables attributed to a principal component, was calculated using the *Promax* method, one of the techniques of oblique rotation. We used oblique rotation because the resultant PCs were likely correlated with other components. Components with an eigenvalue of >1.0 were retained.

## 3. Results

### 3.1. Study Population

**Figure 1:**
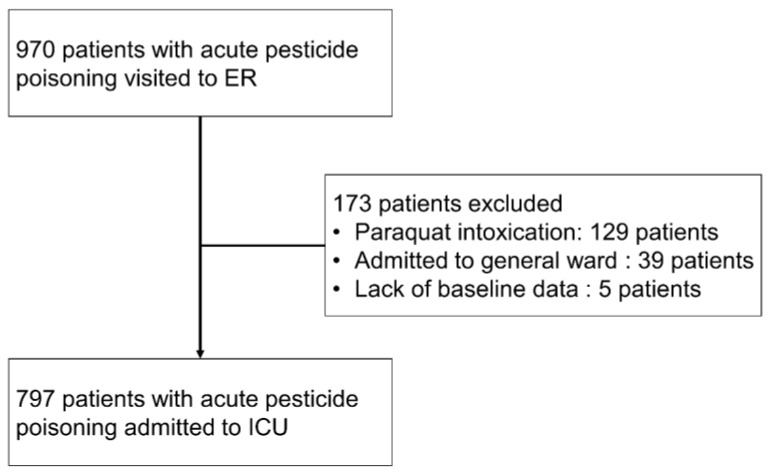
Flow chart showing the inclusion and exclusion of patients in the study.

A total of 970 patients with pesticide intoxication were admitted to our Institute of Pesticide Poisoning between January 2015 and December 2019. Among them, 129 patients were removed due to paraquat intoxication. After exclusion patients who admitted to general ward, we remained 802 patients who admitted to ICU via ER. Overall, five patients were excluded because they lacked baseline data (four had missing HCO_3_^−^ values, and one was missing the initial GCS score). Finally, 797 patients were included in the study (Figure 1).

Baseline demographic, clinical, and biochemical characteristics according to 30 day mortality are summarized in Table 1. Of the 797 patients included in the study, 76 patients (9.5%) died within 30 days. Patients who died within 30 days, referred to as non-survivors, were more likely to be older, have unstable vital signs, and ingest larger amounts of pesticides. The non-survival group had higher APACHE II scores, higher serum lactate and creatinine levels, and lower estimated glomerular filtration rate (eGFR) than the survivor group (Table 1).

### 3.2. Effect of Acid Base Markers on Mortality

The difference in acid-base markers between the two groups is summarized in Table 2. In patients who died within 30 days, pH, HCO_3_^−^, tCO_2_, BE, and SIDe were lower than those who survived more than 30 days. SIDa, SIG, A_Tot_, and corAG were higher in the non-survivors compared to the survivors (Table 2).

Logistic models investigating the association between the parameters assessing acid-base status—based on physiologic, BE-based, and physicochemical approaches—and 30 day mortality are presented in Table 3. The APACHE II score was independently associated with 30 day mortality and it was compared with other variables as a reference in Table 3. Except for pCO_2_, all parameters were associated with 30 day mortality in the univariable logistic model. However, A_Tot_ failed to show an association with 30 day mortality in the multivariable logistic model. Because SIG was calculated by subtracting SIDe from SIDa, it could not be used in the multivariable model concurrently with SIDa and SIDe. Interestingly, higher SIDa and lower SIDe were associated with mortality in a univariable model, whereas the result was paradoxically reversed in the multivariable model (Table 3).

### 3.3. PCA Shows a Formative Construct of Physicochemical Acid-Base Status

**Figure 2:**
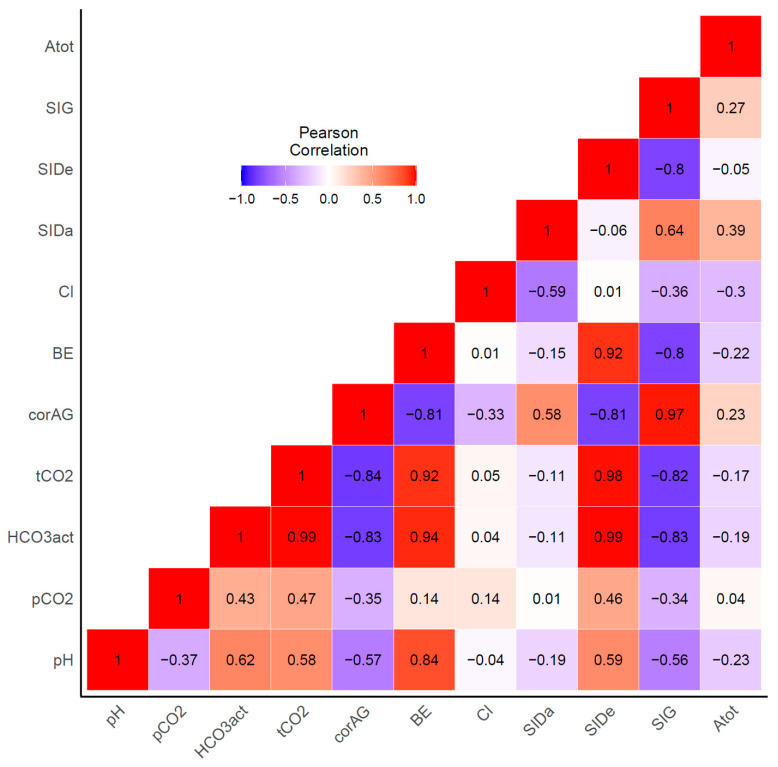
Pearson’s correlation between each parameter assessing acid-base status.

As shown in Figure 2, there were large inter-variable correlations between variables associated with acid-base balance. The HCO_3_^−^ and tCO_2_ concentrations were positively correlated with BE and SIDe, and negatively with corAG and SIG. These correlations between original variables might contribute to the paradoxical reverse observed in the multivariable model described above (positively association in the univariable analysis was inversed to negatively association in the multivariable analysis or vice versa, as observed in Table 3, e.g., odds ratios of SIDa in the univariable was 1.16; however, changed to 0.86 in the multivariable analysis), indicating that the traditional statistical models were inappropriate for our data. To address these intercorrelations between variables, we performed PCA.

**Figure 3:**
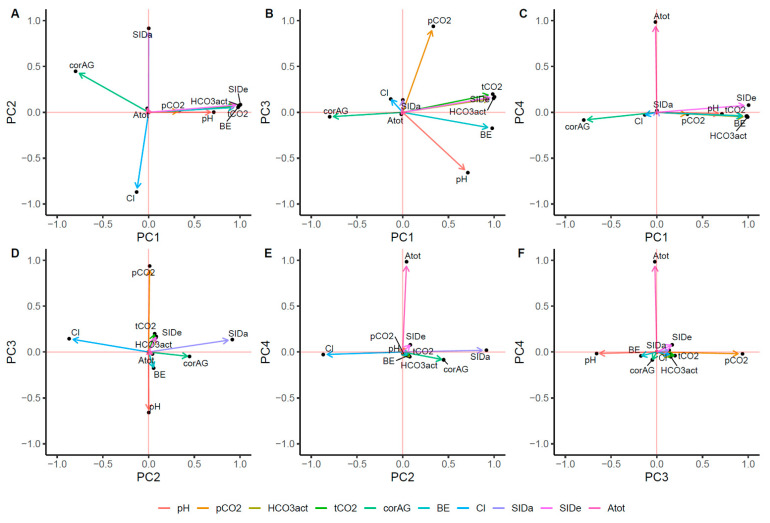
Factor loadings after principal component analysis. The figures representing factor loading in Cartesian coordiates between PC1 and PC2 (**A**), PC1 and PC3 (**B**), PC1 and PC4 (**C**), PC2 and PC3 (**D**), PC2 and PC4 (**E**), and PC3 and PC4 (**F**).

A total of four PCs were selected based on eigenvalues of >1.0. Table 4 shows the eigenvalue of the PCA. Overall, 95% of the total variance could be explained by 4 PCs. Based on the PCA, we interrogated the hidden axes explaining acid-base status (Figure 3). SIDe, HCO_3_^−^, tCO_2_, BE, corAG, and pH were loaded to PC1, the first principal component. PC1 accounted for 52.0% of the total variance. Given the results, we referred to PC1 as the total buffer base. PC2 was associated with SIDa and chloride. PC3 was correlated with pCO_2_, suggesting a respiratory component of the acid-base balance. PC4 was associated with A_Tot_, implying the weak acid’s role in the pathogenesis of acid-base disturbances in patients with pesticide poisoning.

### 3.4. Impact of Principal Components on Mortality

When the variables are reduced to PCs (i.e., reduction in dimension), the inherent information of the principal component would be lost. Therefore, we tested whether these PCs could predict the mortality in acute pesticide poisoning patients as the original variables did. The results from the logistic regression model using PCs are summarized in Table 5. In univariate analysis, each component was associated with mortality within 30 days, whereas, in the multivariate logistic regression model, the significant relationship of PC4 (referred to as concentration of weak acid) with mortality within 30 days was attenuated, suggesting that strong ions played a detrimental role in the death in patients with pesticide poisoning. A paradoxically-reversed odds ratio between SIDa and SIDe, observed in the multivariable model using original variables, was not seen in the multivariable model using PCs.

An increase in PC1 was protective for patients, suggesting both that the capacity of buffers to receive and handle acids was crucial and that intoxicated patients were protected by the total buffer base. An increase in PC2 (SIDa and chloride) and PC3 (pCO_2_) was associated with 30 day mortality, indicating the importance of SID and respiratory failure as risk factors for death in patients with pesticide intoxication.

### 3.5. Differences in Principal Components between Pesticides Categories

**Figure 4:**
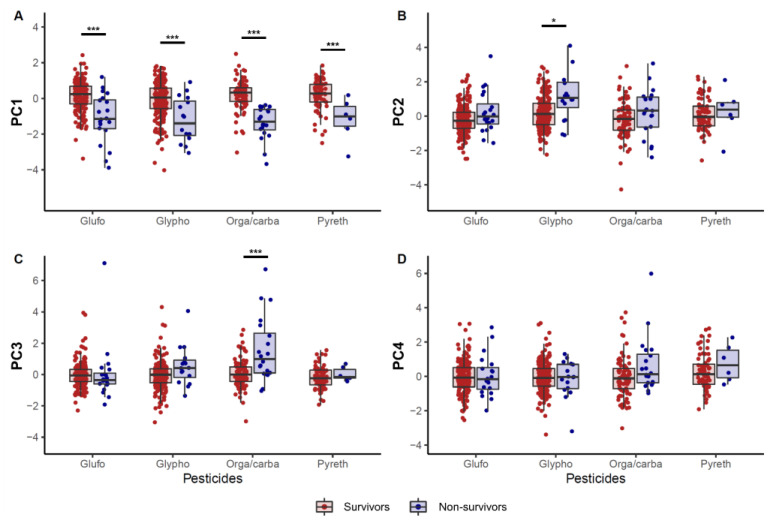
Differences in principal components according to the pesticide category. (**A**) principal component 1, (**B**) principal component 2, (**C**) principal component 3, and (**D**) principal component 4. * *p* < 0.05, *** *p* < 0.001.

The effect of pesticide types on PCA factors was investigated (Figure 4). In the non-survivors, PC1 was lower than in the survivors, irrespective of the ingested pesticide category. PC2 of the non-survivors was higher than that of the survivors in only the glyphosate pesticide category. PC3, representing respiratory components, was higher in non-survivors of organophosphate or carbamate pesticide poisoning. There were no differences in PC4 according to the pesticide category.

## 4. Discussion

Several approaches have been applied to illustrate acid-base physiology, which can be summarized as descriptive, semi-quantitative, and quantitative, representing physiologic, BE-based, and physicochemical approaches, respectively [24], and can be further organized into two perspectives, the traditional and modern perspectives [25]. We showed the significance of these approaches in acute pesticide poisoning. This study also showed not only that various acid-base parameters were significantly different between survivors and non-survivors of acute pesticide poisoning, but also that the acid-base changes after pesticide intoxication could be explained by four PCs, which resembled physicochemical models. Our results also implied the difference in the mode of death according to pesticide categories, including glufosinate, glyphosate, organophosphate or carbamate, and pyrethroid. As a result, patients intoxicated with glufosinate and pyrethroid died by the generation of large amounts of acids. However, in patients with glyphosate intoxication, not only the generation of acids but also increased SID was associated with mortality. Similarly, in the cases of organophosphate or carbamate pesticide poisoning, respiratory failure played a detrimental role additive to those of the accumulated acids.

Drug-induced acid-base disorders can be classified into five different categories based on pathophysiology: (1) metabolic acidosis caused by acid overload, which may occur through the accumulation of acids by endogenous mechanisms; (2) base loss, proximal renal tubular acidosis caused by drugs; (3) alkalosis resulting from acid or chloride loss via renal or extrarenal (e.g., laxative drugs) mechanisms; (4) exogenous bicarbonate loads such as in milk-alkali syndrome, overshoot alkalosis after bicarbonate therapy, or citrate administration; and (5) respiratory acidosis or alkalosis resulting from drug-induced depression of the respiratory center or neuromuscular impairment [26]. Acute pesticide poisoning can affect acid-base status because of the toxicity of ingredients and additives, because commercially used pesticides contain various additives and ingredients, and these usually act as anions, such as sulfate. In the traditional view, metabolic acidosis observed in the non-survivors was accompanied by an increased anion gap. The accompanying decrease across the total buffer base represented by HCO_3_^−^, tCO_2_, BE, and SIDe suggests that an increase in acid was the cause of metabolic acidosis (Table 2).

In clinical practice, the physiological approach is a reasonable tool to predict the prognosis of critically ill patients. Some reports showed that AG was a good prognostic marker in acute pesticide poisoning [3,4,5,7]. Another report suggested that the prognostic significance of SIG was inferior to that of arterial lactate concentrations for critically ill patients [12]. However, the physicochemical approach to acid-base assessment may help identify important acid-base abnormalities that are not apparent using physiological or BE approaches alone [27]. In patients with acute pesticide poisoning, the physicochemical approach might be more predictable because various ingredients attached to pesticides act as biologic solutions and affect other components, including the acid-base balance in the blood. There have been neither reports on acid-base status through a physicochemical approach nor a comparison of the prognosis of acute pesticide poisoning patients according to the three approaches.

Our results showed that all variables associated with acid-base balance, except pCO_2_, might be a risk factor for mortality (Table 3). However, as our institution was a tertiary pesticide intoxication center, many patients in our cohort were transferred via the local ER after intubation due to respiratory failure. These factors could cause bias in our results. Additionally, associations between mortality and SID were complicated to explain. Specifically, the univariable model results were paradoxically reversed in the multivariable model (Table 3). Considering the interactions between each variable (Figure 2), this discrepancy might be explained by each acid-base parameter interacting with the other. Thus, PCA was performed to reduce the dimensionality of the original data [19]. PCs resembled the parameters in the physicochemical approaches. PC1 was positively associated with SIDe, HCO_3_^−^, tCO_2_, BE, pH, and negatively with corAG. Mathematically, SIDe represents the total buffer base and approximates BE, because BE is the total buffer base calculated from the normal bicarbonate and pH values [25]. Therefore, PC1 might be the total buffer base that could compensate for changes in acid-base disturbances, notably increasing acids, which might play a role in attenuating the toxicity of pesticides, as shown in our study. PC2 was associated with SIDa and chloride. PC3 could explain the respiratory component of acid-base balance.

We showed that four PCs generated by PCA could explain the acid-base status related to mortality in acute pesticide poisoning (Figure 4, Table 4). PC1 and PC3 were important risk factors in predicting mortality, and PC2 was to a lesser degree, even though statistically significant in patients with acute pesticide poisoning (Table 4). We also investigated whether some of the PCs were related to mortality according to the classification of pesticide poisoning (Figure 4). Figure 4 shows that all pesticides had lower PC1 in the non-survivors, suggesting that it was related to mortality in all pesticides, which could explain why the circulating total buffer base was important in acute pesticide poisoning as mentioned above. In the organophosphate or carbamate subset, PC3 was higher in the non-survivors, suggesting that the respiratory component could play an important role in acute organophosphate or carbamate poisoning. These results are not only intuitable but also indisputable. Similarly, PC2 was higher in patients with glyphosate poisoning who died within 30 days. Accordingly, our results implied that different effects of pesticides on mortality might exist.

Interestingly, SIDa increased despite metabolic acidosis (Table 2), although metabolic alkalosis is associated with increased SIDa [11]. Of note, higher SIDa was associated with mortality, especially in glyophosate poisoning (Figure 4). SIDa is simply the difference between the activity of all abundant cations and that of all abundant anions. Normally this difference is approximately 39 ± 1 mEq/L. Values lower than 38 mEq/L express metabolic acidosis and values over 40 mEq/L indicate metabolic alkalosis [15]. According to electroneutrality [25], increased SIDa despite metabolic acidosis might be associated with hypocalcemia in non-survivors, as shown both in our study (Table 1) and in previous reports [28,29], or accumulation of non-measured anions, such as sulfate, especially abundant in pesticides. Therefore, it is probably because many anions included in pesticide formula rapidly are absorbed in the body, which is considered a peculiar characteristic of pesticide poisoning. Future research should elucidate this causal relationship.

Our study had some limitations. First, our study had a retrospective design. Second, each pesticide may contain different additives, which could not be considered. Third, the acid-base balance could change over time after acute poisoning, which might influence our results. However, they could not be readily considered in real-world studies based on humans because our study had based on people with suicide attempts. Fourth, an association between cholinesterase levels and the acid-base status was not explored in the cases of organophosphate or carbamate intoxication. It deserved to be evaluated in further study to estimate. Fifth, the effect of other medication was not considered. As illegal drugs such as narcotics are uncommon in Korea, the effect of the illicit drugs could be negligible. Although the medicines for underlying comorbidities could affect the results, it was assumed to have a trivial effect on our result.

## 5. Conclusions

Acid-base abnormalities were associated with mortality in patients with acute pesticide poisoning, and several approaches were closely correlated. The intercorrelation between parameters assessing acid-base status could be analyzed using PCA, irrespective of what approaches (physiologic, BE-based, or physicochemical) were used to describe the acid-base balance. In this way, we revealed that SID, both and apparent and effective, and pCO_2_ among these variables could be important factors to predict mortality in patients with acute pesticide poisoning.

## Figures and Tables

**Table 1:** Baseline characteristics of enrolled patients (*n* = 797).

Variables	Survivors (*n* = 721)	Non-Survivors (*n* = 76)	*p* Value
Age, year	61 (50–75)	74 (63–81)	<0.001
Male, *n* (%)	448 (62.1)	54 (71.1)	0.160
Mean arterial pressure, mmHg	97 (87–107)	89 (63–100)	<0.001
Heart rate, beats/min	88 (77–98)	96 (79–111)	0.001
Respiratory rates, count/min	20 (18–20)	20 (18–22)	0.774
Body temperature, °C	36.4 (36.0–36.9)	36.0 (35.1–36.5)	<0.001
Body mass index, kg/m^2^	22.5 (20.3–24.8)	22.0 (20.2–24.2)	0.303
Current smoking, *n* (%)	258 (35.8)	15 (20.5)	0.013
Alcohol, *n* (%)	351 (48.7)	23 (31.5)	0.007
Hypertension, *n* (%)	264 (36.6)	30 (40.0)	0.651
Diabetes, *n* (%)	130 (18.0)	16 (21.3)	0.585
Respiratory failure, *n* (%)	180 (25.0)	61 (80.3)	<0.001
Pesticides, *n* (%)			0.010
Glufosinate	176 (24.4)	20 (26.3)	
Glyphosate	189 (26.2)	16 (21.1)	
Organophosphate or carbamate	88 (12.2)	20 (26.3)	
Pyrethroid	71 (9.8)	6 (7.9)	
^†^ Others	197 (27.3)	14 (18.4)	
Estimated amount ingested, *n* (%)			0.001
≤50 mL	143 (19.8)	5 (6.6)	
51~100 mL	143 (19.8)	12 (15.8)	
101~200 mL	140 (19.4)	14 (18.4)	
201~300 mL	124 (17.2)	13 (17.1)	
>300 mL	97 (13.5)	23 (30.3)	
Unknown	74 (10.3)	9 (11.8)	
APACHE II, score	8 (5–11)	21 (12–27)	<0.001
Glasgow Coma Scale, score	15 (13–15)	9 (3–14)	<0.001
Hemoglobin, g/dL	14.0 ± 1.9	13.9 ± 2.2	0.640
Hematocrit, %	40.7 ± 5.0	41.4 ± 6.0	0.380
White blood cells, count/mL	10.2 (7.4–14.2)	14.8 (9.6–19.9)	<0.001
Platelet, count/mL	240 (201–291)	235 (200–291)	0.556
Protein, g/dL	7.0 (6.5–7.4)	6.8 (5.8–7.2)	0.002
Albumin, g/dL	4.3 (4.0–4.6)	3.9 (3.5–4.4)	<0.001
Blood urea nitrogen, mg/dL	14.5 (10.9–18.5)	18.2 (14.5–22.4)	<0.001
Creatinine, mg/dL	0.8 (0.7–1.0)	1.1 (0.9–1.5)	<0.001
eGFR, mL/min/1.73 m^2^	89.9 (69.0–105.3)	58.0 (42.0–81.2)	<0.001
Total bilirubin, mg/dL	0.5 (0.3–0.7)	0.5 (0.3–0.7)	0.640
AST, IU/L	26 (21–36)	47 (31–70)	<0.001
ALT, IU/L	18 (13–27)	21 (13–33)	0.391
ALP, IU/L	70 (57–87)	84 (71–100)	<0.001
Uric acid, mg/dL	5.2 (4.1–6.5)	5.7 (4.3–7.1)	0.049
Sodium, mEq/L	142 (139–144)	142 (140–145)	0.249
Potassium, mEq/L	3.9 (3.7–4.3)	4.0 (3.5–4.6)	0.909
Chloride, mEq/L	103 (100–106)	102 (99–104)	0.007
Calcium, mg/dL	9.0 (8.5–9.4)	8.6 (8.0–9.3)	0.006
Phosphate, mg/dL	3.3 (2.8–4.1)	4.5 (3.6–5.7)	<0.001
Lactate, mmol/L	2.4 (1.4–4.0)	5.9 (3.7–9.2)	<0.001
C-reactive protein, mg/L	1.2 (0.5–3.7)	1.8 (0.8–9.4)	0.016

^†^ A detailed representation for the “other” pesticide group was shown in Supplemental Table S1. In patients who were intubated, the verbal GCS score was estimated by the following equation: estimated verbal GCS = (2.3976) × [motor GCS × (−0.9253)] + [eye GCS × (−0.9214)] + [(motor GCS)^2^ × (0.2208)] + [(eye GCS)^2^ × (0.2318)]. 46 patients were missing body mass index values; 4 were missing smoking status; 4 were missing alcohol status; 1 was missing diabetes status; 1 was missing hypertension status; 15 were missing lactate levels. Abbreviations: APACHE, acute physiology and chronic health evaluation; eGFR, estimated glomerular filtration rate; AST, aspartate aminotransferase; ALT, alanine aminotransferase; ALP, alkaline phosphatase.

**Table 2:** Differences in variables associated with acid-base balance and parameters assessing acid-base status in survivors and non-survivors on the first day (*n* = 797).

Acid-Base Marker	Survivors (*n* = 721)	Non-Survivors (*n* = 76)	*p* Value
pH	7.38 (7.33–7.42)	7.25 (7.08–7.35)	<0.001
pCO_2_, mmHg	38 (33–42)	35 (30–44)	0.268
pO_2_, mmHg	85 (73–98)	75 (61–97)	0.001
O_2_ saturation, %	96 (94–97)	93 (86–96)	<0.001
HCO_3_^−^, mEq/L	22.2 (19.3–24.8)	16.0 (12.4–19.2)	<0.001
tCO_2_, mEq/L	23.4 (20.4–26.1)	17.4 (13.5–20.4)	<0.001
BE, mmol/L	−2.3 (−5.5–0.4)	−10.9 (−16.8–−5.2)	<0.001
SIDa, mEq/L	42.5 (40.5–44.5)	44.0 (40.6–48.6)	0.001
SIDe, mEq/L	25.5 (22.4–28.1)	19.7 (16.1–22.9)	<0.001
SIG, mEq/L	16.6 (13.9–20.3)	23.5 (19.4–32.4)	<0.001
A_Tot_, mEq/L	5.2 (4.8–5.7)	5.4 (4.8–6.1)	0.021
corAG, mmol/L	13.8 (11.1–17.5)	23.0 (17.3–29.9)	<0.001

Abbreviations: pH, power of hydrogen; pCO_2_, partial pressure of carbon dioxide; pO_2_, partial pressure of oxygen; HCO_3_^−^, bicarbonate concentration; tCO_2_, total concentration of carbon dioxide; BE, base excess; SIDa, apparent strong ion difference; SIDe, effective strong ion difference; SIG, strong ion gap; A_Tot_, total concentration of serum weak acids; corAG, corrected anion gap.

**Table 3:** Logistic regression models of APACHE II score and parameters assessing acid-base status in predicting mortality in patients with acute pesticide poisoning.

Variables	Univariable		Multivariable	
	Odds Ratio	*p*-Value	Odds Ratio	*p*-Value
APACHE II	1.20 (1.16–1.24)	<0.001	–	–
BE	0.83 (0.80–0.86)	<0.001	0.79 (0.71–0.88)	<0.001
corAG	1.17 (1.14–1.22)	<0.001	1.28 (1.11–1.48)	<0.001
SIDa	1.16 (1.10–1.23)	<0.001	0.86 (0.74–0.99)	0.043
SIDe	0.81 (0.77–0.85)	<0.001	1.42 (1.17–1.73)	<0.001
SIG	1.16 (1.13–1.21)	<0.001	NA	NA
A_Tot_	1.45 (1.11–1.88)	0.005	0.83 (0.60–1.15)	0.277
pCO_2_	1.02 (1.00–1.04)	0.069	–	–

SIG was calculated by subtracting SIDe from SIDa and the three variables could not be input into the multivariable model concurrently. Abbreviations: APACHE, acute physiology and chronic health evaluation; BE, base excess; corAG, corrected anion gap; SIDa, apparent strong ion difference; SIDe, effective strong ion difference; SIG, strong ion gap; A_Tot_, total concentration of serum weak acids; pCO_2_, partial pressure of carbon dioxide; NA, not available.

**Table 4:** Results of principal component analysis.

Variables	PC1	PC2	PC3	PC4
Eigenvalue	5.19	1.82	1.47	1.01
Proportion of variance	0.52	0.18	0.15	0.10
Cumulative variance	0.52	0.70	0.85	0.95
Loadings				
SIDe	1.00			
HCO_3_^−^	0.99			
tCO_2_	0.99			
BE	0.98			
corAG	−0.80	0.45		
pH	0.71		−0.66	
SIDa		0.92		
Cl		−0.87		
pCO_2_	0.34		0.94	
A_Tot_				0.99

When the coefficient equaled to 0.3 or more, factor loading was considered significant. Factor loadings are presented here, after sorting in small orders from large numbers. As strong ion gap (SIG) was calculated by subtracting SIDe from SIDa, SIG was removed from the principal component analysis. When SIG was input with other variables, the inter-item correlation matrix failed to be positive definite. Abbreviations: SIDe, effective strong ion difference; HCO_3_^−^, bicarbonate concentration; tCO_2_, total concentration of carbon dioxide; BE, base excess; corAG, corrected anion gap; pH, power of hydrogen; SIDa, apparent strong ion difference; Cl, chloride; pCO_2_, partial pressure of carbon dioxide; A_Tot_, total concentration of serum weak acids.

**Table 5:** Logistic regression analysis of components predicting mortality in patients with acute pesticide poisoning.

PCs	Univariable		Multivariable	
	Odds Ratio	*p* Value	Odds Ratio	*p* Value
PC1	0.32 (0.25–0.41)	<0.001	0.37 (0.29–0.47)	<0.001
PC2	1.82 (1.46–2.28)	<0.001	1.33 (1.04–1.70)	0.023
PC3	1.67 (1.38–2.04)	<0.001	1.53 (1.24–1.90)	<0.001
PC4	1.33 (1.06–1.65)	0.012	0.93 (0.72–1.18)	0.537

## Data Availability

Data could be available if a reasonable request exists. However, it must be approved by the Institutional Review Board prior to the export of data.

## Supplementary Materials

The following are available online at https://www.mdpi.com/2305-6304/9/2/22/s1, Table S1: A detailed representation of the “other” pesticides group.
