# Supplementary material for: Impact of Acid-Base Status on Mortality in Patients with Acute Pesticide Poisoning
Source: Toxics. 2021 Jan 23;9(2):22. doi: 10.3390/toxics9020022 (PMC7911411; doi:10.3390/toxics9020022)
Supplement: Supplementary file 1 [file toxics-09-00022-s001.pdf]

Supplementary Materials: Impact of Acid-Base Status on Mortality in Patients with Acute Pesticide Poisoning

Hyo-Wook Gil [1], Min Hong [2], HwaMin Lee [2], Nam-jun Cho [1], Eun-Young Lee [1] and Samel Park [1,*]

Table S1. A detailed representation of the "other" pesticides group.

Other Pesticides	Count	Other Pesticides	Count
abamectin	1	fertilizer	4
acequinocyl	1	fungicide	6
acetanilide	1	imidazole	1
acetylaniline	1	kasugamycin	1
adhesive_agent	4	lambda_cyhalothrin	1
alryoxylcarboxide	1	linuron	1
amide	2	lufenuron	2
anilin	1	neonicotinoid	5
antibiotics	11	niacin	3
arsenic	1	oxadiazole	5
aryloxyphenopropionate	1	permethrin	1
benzobicyclon	1	phenoxy	15
benzohydrazide	3	polyoxyethylene methylpolysiloxane	1
bistrufluron	1	pyrol	1
blend of alkalaryl polyethoxylate and sodium salt of alkyl sulfonate alkylate	2	sethoxydim	1
buprofezin	2	sulfonylurea	2
cartal hydrochloride	2	sulfoxaflor	1
chlorfenapyr	2	sulfoxmine	1
chloroacetamide	17	sulfur	1
chloronicotinyl	9	sulfuryl_fluoride	5
chlorophenoxy	1	tetramicacid	3
chlorothalonil	1	tetrazolinone	1
coumarin	15	tetrazolium_oxide	1
diamide	5	tiadinil	1
diazine	1	triazole	2
diethyltoluamide	1	tricyclzaole	2
dinitroaniline	23	urea	6
emamectin	2	unknown	22
endosulfan	2	Total	211
